# Can we improve the detection rate of prostate cancer using standard 12‐core TRUS‐guided prostate biopsy? Focused on the location of prostate biopsy

**DOI:** 10.1002/cam4.2990

**Published:** 2020-04-12

**Authors:** Sangjun Yoo, Jungyo Suh, Juhyun Park, Min Chul Cho, Hwancheol Son, Hyeon Jeong

**Affiliations:** ^1^ Department of Urology Seoul National University Boramae Medical Center Seoul Korea; ^2^ Seoul National University Seoul Republic of Korea

**Keywords:** biopsy, prostate, prostatectomy, prostatic neoplasms, ultrasonography

## Abstract

**Background:**

We assessed the effect of biopsy location on the prostate cancer detection and clinically significant prostate cancer.

**Methods:**

A total of 2774 patients with 12‐core prostate transrectal ultrasound‐guided prostate biopsy were included for per core analysis. Multivariate Cox regression analysis was performed to evaluate the effect of the location of biopsy on the prostate cancer and clinically significant prostate cancer detection.

**Results:**

Prostate cancer was found in 775 patients (27.9%) and 576 prostate cancer patients (20.8%) were found to be clinically significant. The core length (*P* = .043), tumor length (*P* < .001), and % tumor length (*P* < .001) were significantly different according to the biopsy location. The detection rates for prostate cancer and clinically significant prostate cancer differed significantly according to the location of biopsy. Multivariate analysis revealed that the apical core was significantly related with increased detection of prostate cancer and clinically significant prostate cancer. The lateral core, in addition to apical core, was found to be significantly related with increased detection rates of prostate cancer and clinically significant prostate cancer in men with prostate‐specific antigen <10 ng/mL.

**Conclusions:**

More in‐depth discussions on the location of standard 12‐core prostate biopsy are considered necessary. Apical core and lateral core biopsies may be helpful, especially in patients with prostate‐specific antigen ˂10 ng/mL if additional biopsies are planned following findings of no target lesions on imaging studies.

## Graphical abstract

More in‐depth discussions on the location of standard 12‐core prostate biopsy are considered necessary. Apical core and lateral core biopsies may be helpful, especially in patients with prostate‐specific antigen levels ˂10 ng/mL if additional biopsies are planned following findings of no target lesions on imaging studies.

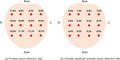

## 1 INTRODUCTION

Prostate biopsy techniques have been greatly modified with improvements in prostate imaging modalities, which include magnetic resonance imaging (MRI).^1^, ^2^, ^3^ Based on the Prostate Imaging‐Reporting and Data System version 2 introduced in 2015,^4^ the detection rate of prostate cancer (PC) using MRI‐fusion biopsy was reported to be similar to that using 12‐core transrectal ultrasound (TRUS)‐guided prostate biopsy with a low number of biopsied cores.^5^, ^6^ A recent prospective study reported that using MRI‐fusion biopsy and standard 12‐core TRUS‐guided biopsy together yielded superior detection rates of PC than when only 1 of the biopsy techniques is used.^3^

Many studies reported that MRI‐fusion biopsy shows a higher detection rate of clinically significant prostate cancer (csPC) than TRUS‐guided biopsy.^5^, ^7^ Due to the widespread use of active surveillance in low‐risk PC patients,^8^ the importance of CSPC detection has increased like never before. Therefore, targeted biopsies based on imaging studies such as MRI fusion biopsy are becoming more common, especially for men with negative results on previous biopsy.^9^

Despite the remarkable advancements in prostate imaging and biopsy techniques, standard 12‐core TRUS‐guided biopsy is still regarded as the gold standard methods for diagnosing PC in biopsy‐naive patients with suspected PC.^10^ As mentioned above, a recent prospective study reported that MRI fusion biopsy combined with standard TRUS‐guided 12‐core biopsy had detection rates of PC superior to MRI fusion biopsy alone even with the improvements in imaging modalities.^3^ In addition, due to the reported detection of a non‐negligible number of csPC cases with no visible lesions on MRI using 12‐core TRUS‐guided biopsy,^11^ it is unlikely that the role of this biopsy technique will wane in the near future. However, to keep up with recent advancements, the standard 12‐core TRUS‐guided prostate biopsy technique needs further improvement in terms of the detection rate of PC, especially csPC.

Based on the shape and zonal anatomy of the prostate, we hypothesized that the detection rates of PC and csPC may vary according to the biopsied location. We therefore assessed the effects of prostate biopsy location on the PC and csPC detection rates using per core analysis. Furthermore, as the detection of csPC is considered particularly important for men with serum prostate‐specific antigen (PSA) ˂10 ng/mL, we also investigated the effects of biopsy location on the detection of PC and csPC in men with serum PSA ˂10 ng/mL.

## 2 MATERIALS AND METHODS

### 2.1 Study population

A total of 2824 patients who underwent 12‐core TRUS‐guided transrectal prostate biopsy at Boramae Medical Center from 2003 to 2014 were initially selected. Of these, 30 patients with less than 12 biopsy cores and 20 patients with additional target biopsy were excluded. A final total of 2774 patients who underwent 12‐core TRUS‐guided biopsy without additional target biopsy were selected for the analysis. The medical records of these patients were reviewed, retrospectively. The institutional review board of our institute approved this study.

### 2.2 Patient evaluation

Prostate biopsy is generally recommended in our institute to patients with serum PSA ≥ 3 ng/mL. Before the biopsy, the prostate volume is measured and radiologists specialized in urology check for hypoechoic lesions. MRI was not performed before prostate biopsy because the Korean national health insurance system does not cover costs for MRI procedures performed before histological diagnosis of PC. Transrectal prostate biopsy was performed using the standard 12‐core biopsy technique. Specimens obtained from prostate biopsy were examined by pathologists specialized in urology and Gleason scores were categorized according to the 2014 International Society of Urological Pathology classification.^12^ The total core length and tumor length on each biopsy core were also assessed by pathologists. The % tumor length was calculated using the following formula: % tumor length = tumor length/total core length × 100.

### 2.3 Outcomes

The primary outcomes were the detection rates of PC and csPC using 12‐core prostate biopsy on per core analysis. In this study, csPC was defined as patients with PC who did not meet the Prostate Cancer Research International Active Surveillance (PRIAS) criteria.^13^

### 2.4 Statistical analysis

Patient characteristics were expressed as mean ± standard deviation (SD), median with interquartile range (IQR), or number with percentage. For per core analysis, location of biopsy core was divided into two lateral categories (lateral and medial) and three axial categories (apex, mid, and base). As prebiopsy serum PSA is the most valuable predictive factor for the PC detection, and aggressiveness, sub‐analysis was performed after selecting patients with serum PSA ˂10 ng/mL. Biopsy characteristics, including, biopsied core length, tumor length, and % tumor length, were also assessed and compared according to the location of biopsied cores using Student's *t* test*.* In addition, the PC and csPC detections were compared according to the location of biopsied cores using Pearson's chi‐square test. We performed univariate and multivariate Cox regression analyses to assess the effects of biopsy location on the detection of PC and csPC at the location of the biopsy core after adjusting other clinical variables. Variables with *P* values of <0.2 in the univariate analysis were selected for the multivariate analysis and backward elimination methods were used for multivariate analysis. All statistical analyses were performed using IBM SPSS Statistics version 21 (IBM SPSS) and *P* values of < .05 were considered statistically significant.

## 3 RESULTS

The mean age of patients included in this study was 66.8 years and the median PSA level was 7.3 ng/mL (Table 1). The mean prostate volume was 44.0 mL and hypoechoic lesions were detected on TRUS in 453 patients (16.3%). Of the 2774 men who underwent standard 12‐core TRUS‐guided prostate biopsy, 775 (27.9%) were found to have PC and 576 (20.8%) were found to have csPC. The Gleason scores of the patients with PC were as follows: 1 in 283 patients (36.5%), 2 in 148 patients (19.1%), 3 in 115 patients (14.8%), 4 in 151 patients (19.5%) and 5 in 78 patients (10.1%).

**Table 1:** Baseline patient characteristics

	Total
Number of patients, n	2774
Age, mean ± SD	66.8 ± 8.2
Body mass index, kg/m^2^, mean ± SD	24.0 ± 2.9
Hypertension, n (%)	1183 (42.6)
Diabetes, n (%)	444 (16.0)
PSA level, ng/mL, median (IQR)	7.3 (4.7‐12.1)
PSA density, ng/mL/cc, median (IQR)	0.18 (0.12‐0.30)
Prostate volume, cc, mean ± SD	44.0 ± 22.7
Hypoechoic lesion on TRUS, n (%)	453 (16.3)
Men with prostate cancer, n (%)	775 (27.9)
Number of positive cores, n, median, (IQR)	3 (1‐6)
Clinically significant prostate cancer, n (%)	576 (20.8)

Core length was significantly longer in apical (*P* = .043) and medial biopsy cores (*P* < .001) (Table 2). Tumor length (*P* < .001) and % tumor length (*P* < .001) were significantly longer in basal biopsy cores. Per core analysis revealed that the detection rates of PC and csPC from biopsies taken from the medial area of the prostate were significantly lower in basal cores compared to apical core in all the patients and in patients with serum PSA levels <10 ng/mL (Table 3). The detection rates of PC and csPC from biopsies taken from the basal area of the prostate were significantly higher in lateral core than in medial core.

**Table 2:** Biopsy related characteristics according to the location of prostate biopsy

	Medial			Lateral			*P* (Medial vs lateral)	*P* (apex vs mid vs base)
	Apex	Mid	Base	Apex	Mid	Base		
Core length, cm, mean ± SD	1.50 ± 0.26	1.46 ± 0.26	1.46 ± 0.35	1.43 ± 0.27	1.43 ± 0.26	1.43 ± 0.24	<.001	.043
Tumor length. cm, mean ± SD	0.57 ± 0.42	0.63 ± 0.42	0.66 ± 0.44	0.55 ± 0.41	0.61 ± 0.44	0.62 ± 0.44	.209	<.001
% tumor length, %, mean ± SD	38.7 ± 29.2	44.2 ± 30.0	46.4 ± 31.3	40.2 ± 32.0	44.0 ± 31.0	44.6 ± 31.4	.897	<.001

**Table 3:** Prostate cancer detection rate according to the location of prostate biopsy

	Prostate cancer			Clinically significant prostate cancer		
	Medial	Lateral	*P*	Medial	Lateral	*P*
(A) Total patients (n = 2774)						
Apex						
Right lobe	309 (11.1)	301 (10.9)	.292	237 (8.5)	248 (8.9)	.896
Left lobe	324 (11.7)	297 (10.7)		254 (9.2)	239 (8.6)	
Mid						
Right lobe	276 (10.0)	295 (10.6)	.288	229 (8.3)	244 (8.8)	.432
Left lobe	277 (10.0)	292 (10.5)		227 (8.2)	235 (8.5)	
Base						
Right lobe	244 (8.8)	305 (11.0)	.002	206 (7.4)	249 (9.0)	.016
Left lobe	237 (8.5)	272 (9.8)		191 (6.9)	216 (7.8)	
*P*	<.001	.810		.004	.752	
(B) Patients with PSA < 10 ng/mL (n = 1866)						
Apex						
Right lobe	117 (6.3)	114 (6.1)	.699	77 (4.1)	90 (4.8)	.255
Left lobe	111 (6.0)	106 (5.7)		75 (4.0)	82 (4.4)	
Mid						
Right lobe	82 (4.4)	111 (6.0)	.008	62 (3.3)	85 (4.6)	.016
Left lobe	88 (4.7)	110 (5.9)		68 (3.6)	86 (4.6)	
Base						
Right lobe	64 (3.4)	107 (5.7)	<.001	49 (2.6)	84 (4.5)	<.001
Left lobe	66 (3.5)	101 (5.4)		46 (2.5)	74 (4.0)	
*P*	<.001	.774		.001	.682	

Multivariate analysis revealed that apical biopsy (Apex, reference; Mid, odds ratio [OR]: 0.904, *P* = .053; Base, OR: 0.804, *P* < .001) was associated with increased detection rate of PC as were age, body mass index, presence of hypertension and diabetes, PSA level, prostate volume, and finding of hypoechoic lesions on TRUS (Table 4). Furthermore, apical biopsy (Apex, reference; Mid, OR: 0.940, *P* = .248, Base; OR: 0.837, *P* = .001) was found to be associated with increased detection rate of csPC in addition to other variables. In patients with serum PSA levels ˂10 ng/mL, apical biopsy (Apex, reference; Mid, OR: 0.843, *P* = .032; Base, OR: 0.697, *P* < .001) and lateral biopsy (OR: 1.307, *P* < .001) was associated with increased PC detection in addition to other variables. Moreover, apical biopsy (Apex, reference; Mid, OR: 0.919, *P* = .326; Base, OR: 0.755, *P* = .002) and lateral biopsy (OR: 1.381, *P* < .001) were associated with increased detection of csPC. However, prostate biopsy location was not associated with csPC with PSA levels ≥ 10 ng/mL (Table S1).

**Table 4:** Impact of location of prostate biopsy on the detection of prostate cancer

	Prostate cancer		Clinically significant prostate cancer	
	OR (95% CI)	*P*	OR (95% CI)	*P*
**(A) Total patients**				
Age (continuous)	1.062 (1.056‐1.069)	<.001	1.065 (1.059‐1.072)	<.001
Body mass index (continuous	1.029 (1.014‐1.044)	<.001	1.033 (1.017‐1.048)	<.001
Hypertension (yes vs n	1.194 (1.091‐1.306)	<.001	1.197 (1.091‐1.312)	<.001
Diabetes (yes vs no	1.214 (1.086‐1.357)	<.001	1.214 (1.083‐1.361)	<.001
PSA level (continuous)	1.022 (1.021‐1.024)	<.001	1.023 (1.022‐1.025)	<.001
Prostate volume (continuous)	0.963 (0.960‐0.966)	<.001	0.960 (0.958‐0.963)	<.001
Hypoechoic lesion on TRUS (yes vs no)	1.927 (1.752‐2.119)	<.001	1.995 (1.811‐2.198)	<.001
Axial location of prostate biopsy				
Apex	Reference		Reference	
Mid	0.904 (0.815‐1.001)	.053	0.940 (0.845‐1.044)	.248
Base	0.804 (0.724‐0.893)	<.001	0.837 (0.751‐0.932)	.001
Sagittal location of prostate biopsy (lateral vs medial)	1.079 (0.991‐1.175)	.081	1.090 (0.998‐1.190)	.055
**(B) Patients with PSA < 10ng/mL**				
Age (continuous)	1.056 (1.047‐1.066)	<.001	1.059 (1.049‐1.070)	<.001
Hypertension (yes vs no)	1.202 (1.049‐1.377)	.008	1.226 (1.060‐1.417)	.006
Diabetes (yes vs no)	1.378 (1.163‐1.633)	<.001	1.417 (1.183‐1.697)	<.001
PSA level (continuous)	1.210 (1.172‐1.249)	<.001	1.249 (1.206‐1.292)	<.001
Prostate volume (continuous)	0.951 (0.946‐0.956)	<.001	0.939 (0.934‐0.945)	<.001
Hypoechoic lesion on TRUS (yes vs no)	2.211 (1.901‐2.571)	<.001	2.493 (2.126‐2.924)	<.001
Axial location of prostate biopsy				
Apex	Reference		Reference	
Mid	0.843 (0.721‐0.986)	.032	0.919 (0.777‐1.087)	.326
Base	0.697 (0.592‐0.820)	<.001	0.755 (0.634‐0.900)	.002
Sagittal location of prostate biopsy (lateral vs medial)	1.307 (1.144‐1.492)	<.001	1.381 (1.198‐1.592)	<.001

## 4 DISCUSSION

As earlier mentioned, prostate biopsy techniques have gradually evolved to minimize the risk of complications and increase the detection rate of csPC.^1^, ^2^, ^3^, ^14^ Even with the recent introduction and wide acceptance of MRI fusion prostate biopsy in clinical practice, its routine performance should be critically considered and its use limited to men with negative results on previous biopsy.^15^ In the current clinical guidelines, standard 12‐core TRUS‐guided biopsy is still regarded as the gold standard methods in men without prior prostate biopsies.^10^ Moreover, a considerable number of studies still dispute that standard 12‐core prostate biopsy and MRI‐fusion biopsy used together have superior detection rates of csPC compared to those of MRI‐fusion biopsy alone.^16^ However, although prostate biopsy techniques have improved significantly in recent times, the standard 12‐core biopsy technique has not changed in over a decade. To optimize the standard 12‐core biopsy technique, the number of biopsies^1^ and other biopsy‐related factors as well as the interpretation of biopsy results^17^ need to be carefully considered. Although an earlier study evaluated the effects of biopsy location on PC detection,^18^ to the best of our knowledge, this is the first study to provide a reasonable and reliable in‐depth analysis of the effect of biopsy location on the detection rates of not only PC, but also csPC.

Our study showed detection rates of 27.9% and 20.8% for PC and csPC, respectively, using 12‐core biopsy, which are similar to the findings of an earlier study.^19^ The detection rates of PC and csPC in men with serum PSA ˂10 ng/mL were 20.9% and 13.2%, respectively, which are also similar to those reported by an earlier study.^20^ However, the detection rates of PC and csPC in this study differed considerably according to biopsy location and apical/ lateral cores showed higher detection rates of PC and csPC, which are consistent with the reports of an earlier study.^18^ Interestingly, apical biopsy was found to be a factor related to increased detection rates of PC and csPC. Moreover, apical biopsy and lateral biopsy were found to be associated with increased PC and csPC detection rates in men with serum PSA levels ˂10 ng/mL after adjusting other variables.

We hypothesized that our findings may be due to anatomical characteristics of the prostate even though we did not assess the reason behind our findings. First, the apex of the prostate is narrow compared to its base. This may account for the higher detection rate of PC in the apical area compared to the basal area, which may be due to a higher percentage of tumor volume in the apex even with comparable PC volumes in the apical and basal areas. Second, the peripheral zone, which is a common site of PC, is normally located in the whole proportion of the prostate apex^21^ with the transitional zone located in the mid and basal prostate. Similarly, the lateral biopsied core may contain a higher proportion of tissue from the peripheral zone than the medial biopsied core as the transitional and anterior fibromuscular zones are located in the medial part of the prostate. However, as described in the results section, no significant differences were observed in the detection rates of PC in men with PSA levels ≥ 10 ng/mL according to the biopsy location, which may be due to large cancer volumes covering considerably large sections of the prostate.

Based on these results and prostate anatomy, biopsies focused on the apical and lateral areas of the prostate need to be considered if there are plans to perform additional biopsies in men with no visible target lesions on MRI and/or prebiopsy MRI. On the basis of our study findings, it is imperative that more reliable studies on the optimal prostate biopsy location are conducted. Although a large number of studies on the optimization of TRUS‐biopsy techniques have been published,^19^, ^22^, ^23^ most of them are outdated and focused only on the number of biopsies. However, based on this study, in addition to the number of biopsied cores, the biopsy location may be key factors influencing the detection of PC and csPC.

Our study results may be useful for clinicians even in this era of MRI fusion biopsy as the per‐patient base sensitivity and specificity for PC detection using MRI were reported to be about 80% in patients with PSA ˂10 ng/mL.^24^ In other words, a non‐negligible number of PC cases cannot be accurately identified even with multiparametric MRI performed prior to prostate biopsy. Moreover, additional apical and lateral core biopsies may be reliable options for detecting hidden PC, including csPC, in patients with previous negative biopsy results and no suspicious lesions on MRI.

However, our study results need to be further validated by studies because other clinical variables remain powerful predictors of the PC and csPC detection. The effects of prostate biopsy location also need to be confirmed in Western patients because PC aggressiveness and prostate volume, which may affect outcomes and the importance of biopsy location, were reported to differ significantly across ethnicities.^25^ This study has several limitations, which include its retrospective design, the small size of the study population, and the long duration of patient enrollment. In addition, the effects of number of prior prostate biopsy, which cannot be assessed in this study, remained to be adjusted in the future study. Despite these limitations, the study results may be considered inspiring for the improvement of the standard 12‐core prostate biopsy technique and useful for taking additional cores in men with no visible lesions on MRI or prebiopsy MRI.

In conclusion, this study showed that apical biopsy and lateral biopsy are associated with increased detection of PC and csPC, especially in men with PSA levels ˂10 ng/mL. Based on these results, more in‐depth discussions on the location of biopsied cores during 12‐core standard TRUS‐guided biopsies are necessary. It may be helpful to take more cores from the apical and lateral areas of the prostate if additional biopsies are planned following findings of no target lesions on imaging studies.

## CONFLICT OF INTERESTS

Authors disclose no financial and personal relationship related to this study.

## AUTHOR CONTRIBUTION

Sangjun Yoo: Drafting; Jungyo Suh: Data collection; Juhyun Park: Statistical analysis; Min Chul Cho: Critical revision; Hwancheol Son: Critical revision; Hyeon Jeong: Conception.

## Supporting information

Table S1
