# Supplementary material for: Can we improve the detection rate of prostate cancer using standard 12‐core TRUS‐guided prostate biopsy? Focused on the location of prostate biopsy
Source: Cancer Med. 2020 Apr 12;9(11):3758–64. doi: 10.1002/cam4.2990 (PMC7286467; doi:10.1002/cam4.2990)
Supplement: Supplementary file 1 — Table S1 [file CAM4-9-3758-s001.docx]

Supplementary Table. Multivariable analysis for predicting clinically significant prostate cancer in men with PSA level ≥ 10 ng/mL

PSA level ≥ 10 ng/mL

OR (95% CI) p

Age (continuous) 1.047 (1.039-1.055) <0.001

Body mass index (continuous) 1.070 (1.050-1.092) <0.001

Hypertension (yes vs. no) 1.135 (1.007-1.279) 0.038

PSA level (continuous) 1.014 (1.012-1.015) <0.001

Prostate volume (continuous) 0.965 (0.962-0.968) <0.001

Hypoechoic lesion on TRUS (yes vs. no) 1.644 (1.450-1.865) <0.001
